# *In Vivo* Detection of the Effect of Electroacupuncture on “Zusanli” Acupoint in Rats with Adjuvant-Induced Arthritis through Optical Coherence Tomography

**DOI:** 10.1155/2016/2681463

**Published:** 2016-11-17

**Authors:** Huiqing Zhong, Hui Yang, Yan Zhou, Zhouyi Guo, Xiuli Wu, Chengkang Su, Jia Long, Jin Lin, Xuemei Jiang

**Affiliations:** ^1^SATCM Third Grade Laboratory of Chinese Medicine and Photonics Technology, College of Biophotonics, South China Normal University, Guangzhou 510631, China; ^2^Infinitus (China) Company Ltd., Guangzhou 510665, China

## Abstract

This study aimed to investigate the effect of electroacupuncture (EA) treatment through optical coherence tomography (OCT)* in vivo* on rats with adjuvant-induced arthritis. OCT images were obtained from the ankle of the right hind paws of the rats in control, model, and EA groups before modelling and 1 day, 8 days, 15 days, 22 days, and 29 days after modelling. Results demonstrated that the OCT signal of the ankle of the right hind paws of the rats was indistinct compared to 1 day after modelling and before modelling in the EA group. In the EA group, the light averaged attenuation coefficients of the ankle tissues decreased as treatment duration was prolonged after EA was administered (3.43, 2.96, 2.61, 2.42, and 2.29 mm^−1^, resp.). There was a significant difference in attenuation coefficient decrease between the 29th d and the 1st d for EA group compared with control group (*P* < 0.01). This condition indicated that the light absorption of the ankle of the treated rats in the EA group decreased. Therefore, OCT can be used to monitor the effect of treatment on rats with arthritis* in vivo*.

## 1. Introduction

Complete Freund's adjuvant is used to induce inflammatory pain in animal models of diseases, such as arthritis. It is often utilised to investigate pain mechanisms [1–3], pain relief [4], and drug-induced anti-inflammatory effects and to explain physical acupuncture methods [5–10]. The effects of treatments on arthritis have been detected by direct observation through the eyes or by measuring circumference* in vivo*. However, these methods fail to detect the change in the depth of arthritis.

OCT is an optical technique used to determine the backscattering of near-infrared light in tissues. OCT is a recently developed noninvasive imaging technique that provides high-resolution cross-sectional images of biological tissue microstructures [11–13]. This technique combines the advantages of interferometers and confocal microscopes to probe weakly backscattered photons from microstructures beneath tissue surfaces. OCT has been successfully applied* in vivo* in medical imaging and diagnostics, such as ophthalmic [14, 15], dermatological [16–18], eye [19], glucose [20], and oral [21–25] treatments.

In this study, OCT was employed to detect the effect of electroacupuncture (EA) treatment on rats. This study aimed to monitor the* in vivo* effect of treatment on rats with arthritis through OCT.

## 2. Materials and Methods

### 2.1. Animals

Forty-five male and female Wistar rats aged 8–10 weeks and weighing 190 ± 10 g were obtained from the Laboratory Animal Centre, Southern Medical University. All experiments were performed under protocols approved by the South China Normal University Animal Care and Use Committee. The rats were housed at a controlled temperature (23 ± 2°C), habituated* ad libitum*, and maintained at 12 h/12 h light/dark cycle. All of the rats were habituated at the laboratory room for at least 1 week before the experiment began. They were then divided randomly into three groups: (1) control group (*n* = 15), which was not treated; (2) model group (*n* = 15), which was not treated after the model was established; and (3) electroacupuncture group (*n* = 15), which was treated with right lateral “Zusanli” electroacupuncture (ST36; ST36 acupoint is located between the tibia and the fibula [26]) after the model was established. Each animal in the model and electroacupuncture groups was injected with 0.1 mL of complete Freund's adjuvant (CFA, Sigma, USA) into the ankle joint of the right hind paw. The CFA injection immediately caused local inflammation, paw swelling, and pain. These symptoms indicated that the model was successfully established.

### 2.2. Electroacupuncture Treatment

From 1 day after modelling, stainless steel acupuncture needles with a diameter of 0.25 mm were inserted into the ST36 acupoint of the right hind paw ipsilaterally at a depth of 7 mm. Continuous-wave stimulation was selected at a frequency of 5 Hz and intensity of 2 mA (6805-AII,* Shantou* Medical Equipment Factory Co., Ltd.) [27–29]. An individual EA session was administered daily for 20 min for 7 days, with 2-day rest for the course of treatment, for 4 consecutive weeks.

### 2.3. OCT System and Analysis of OCT Signal Attenuation

The OCT system used in this study mainly consisted of resource, fibre conduction, reference arm, and sample arm modules. A schematic of the OCT system is shown in a previous study [25]. The broadband light source was a superluminescent diode with a central wavelength of 1310 nm and a bandwidth of 50 nm. The light source yields an axial resolution of 15 *μ*m in a free space. The transverse resolution of the system was approximately 20 *μ*m, as determined by the focal spot size produced by the probe beam. The signal-to-noise ratio of this system was measured at 100 dB. A visible light source (*λ* = 632 nm) was used to guide the probe beam. The OCT system operation was controlled automatically by using a computer [25, 30, 31]. The right ankle joints of the rats in each group were monitored at different time points (0, 1, 8, 15, 22, and 29 days) through OCT.

OCT imaging is based on the difference in the backscattering of light. In this model, light that scattered with the decay of the OCT signal with a depth function is in accordance with Beer–Lambert law. According to Beer–Lambert law, light attenuation inside tissues is exponential. The total attenuation coefficient is *μ*_t_ = *μ*_*s*_ + *μ*_*a*_, where *μ*_*s*_ is the scattering coefficient and *μ*_*a*_ is the absorption coefficient. Only the backscattered components from tissues contribute to the OCT image because *μ*_*a*_ ≪ *μ*_*s*_ for tissues in the NIR spectral range [32–34]. In brief, the differences in the attenuation coefficient in each group can potentially be detected with the OCT system from *μ*_t_. The following expression is a Liebenberg–Marquardt curve fitting algorithm [25, 34]:

(1) $$\begin{array}{c} y=A\exp\left(\;-\mu_{\text{t}}x\;\right)+y_{0}, \end{array}$$

where *y* is the signal, *x* is the penetration depth of the OCT images, and *μ*_t_ is the attenuation coefficient.

### 2.4. Statistical Analysis

Data were presented as means ± SD for a number of sample animals and analysed through paired *t*-test by using SPSS 18.0. *P* < 0.05 indicated significant difference.

## 3. Results and Discussion

### 3.1. Photos

**Figure 1:**
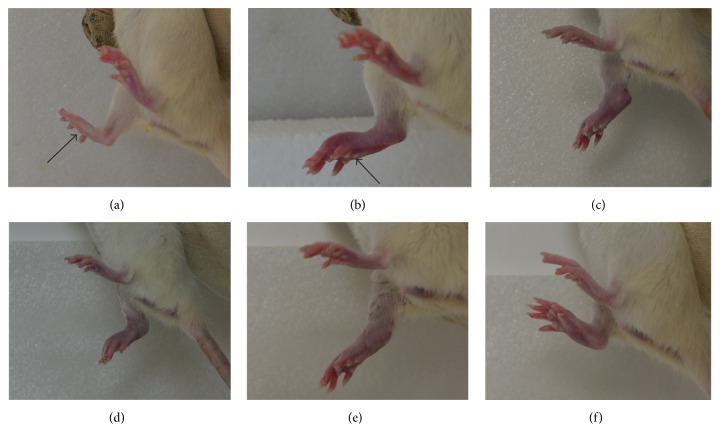
Representative photos of paws of mice in the model group before modelling (a), 1 day after modelling (b), 8 days after modelling (c), 15 days after modelling (d), 22 days after modelling (e), and 29 days after modelling (f). The* black arrow* is the location of injecting complete Freund's adjuvant.

**Figure 2:**
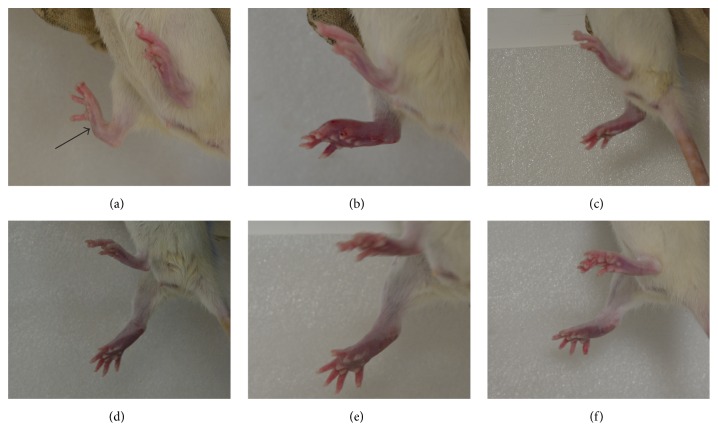
Representative photos of paws of mice in the electroacupuncture group before modelling (a), 1 day after modelling (b), 8 days after modelling (c), 15 days after modelling (d), 22 days after modelling (e), and 29 days after modelling (f). The* black arrow* is the location of injecting complete Freund's adjuvant.

The rats in the control group were not treated, in which no change was observed in the hind paw (not shown). The rats' hind paws in the model group before modelling, 1 day after modelling, 8 days after modelling, 15 days after modelling, 22 days after modelling, and 29 days after modeling are shown in Figure 1. Comparing Figure 1(b) with Figure 1(a), we found that the swelling in the right hind paw was different. By contrast, this finding is not observed in the control group. This result implied that the model was successfully established. After the model was created, the right hind paw could slowly self-heal. However, the effect was not distinct. In some instances, the swelling in the right hind paw may become serious (comparing Figure 1(e) with Figure 1(d)). The rats' hind paws in the electroacupuncture group before modelling, 1 day after modelling, 8 days after modelling, 15 days after modelling, 22 days after modelling, and 29 days after modeling are shown in Figure 2. From 1 day after modelling, the rats in the electroacupuncture group were treated with electroacupuncture for 20 min. It was observed that the swelling in the right hind paw was different (Figures 2(b) and 2(a)). After the treatment time was prolonged, the swelling in the right hind paws of the rats was relieved. On day 29, the swelling in the right hind paws of the rats almost disappeared (Figure 2(f)). This condition indicated that electroacupuncture could stimulate the* “Zusanli”* acupoint to treat the adjuvant-induced arthritis.

### 3.2. Analysis of OCT Signal Attenuation

From the OCT images of the ankle of the right hind paws of the rats in the control group at different times* in vivo*, it can be found that their surface layers of the normal ankle of the right hind paws of the rats are visible with a quite distinct layer structure (not shown).

**Figure 3:**
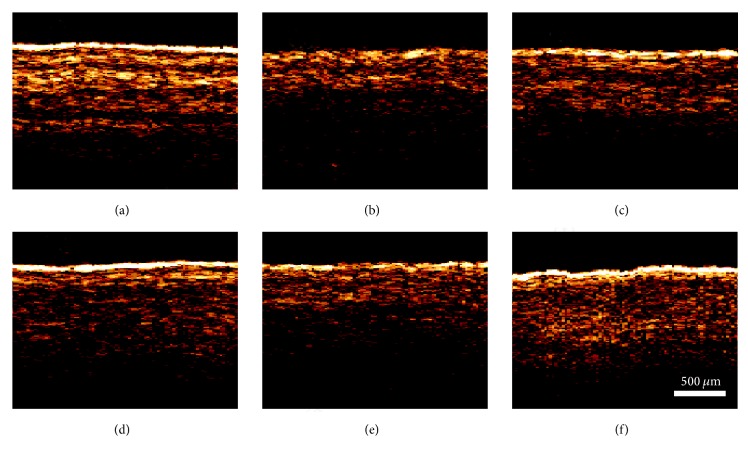
OCT images of the ankle of the right hind paws of the rats in the model group before modelling (a), 1 day after modelling (b), 8 days after modelling (c), 15 days after modelling (d), 22 days after modelling (e), and 29 days after modelling (f). The* white scale* in the image indicates the contrast scales.

**Figure 4:**
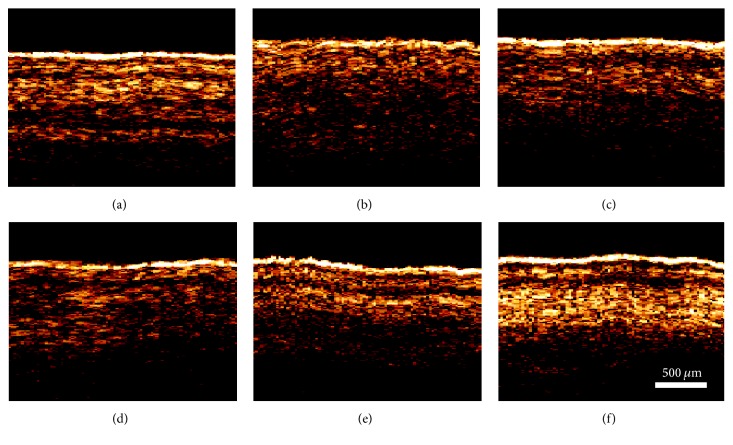
OCT images of the ankle of the right hind paws of the rats in the electroacupuncture group before modelling (a), 1 day after modelling (b), 8 days after modelling (c), 15 days after modelling (d), 22 days after modelling (e), and 29 days after modelling (f). The* white scale* in the image indicates the contrast scales.

However, the layer is difficult to be distinguished because it is injected with 0.1 mL complete Freund's adjuvant in the ankle joint of the right hind paw with swelling (Figure 3(b)). Thus, the OCT signal of the ankle of the right hind paws of the rats shows significant attenuation. This result is in accordance with Figure 1(b). This condition confirms that complete Freund's adjuvant can decrease the OCT signal of local tissues when the adjuvant is locally injected in tissues. This decrease occurs because the rats were not treated in every way after the model was established. Thus, the OCT signals in the ankle of the right hind paws of the rats are also structurally indistinct when the treatment time was prolonged after the model was established (Figures 3(b)–3(f)). The OCT images of the ankle of the right hind paws of the rats in the electroacupuncture group before modelling, 1 day after modelling, 8 days after modelling, 15 days after modelling, 22 days after modelling, and 29 days after modelling are shown in Figure 4. In Figure 4(b), the OCT signal of the ankle of the right hind paws of the rats is indistinct. However, a clear structure is shown (Figure 4(a)). This condition indicated that the model was successfully established. In the electroacupuncture group, from the second day (1st d), the rats are treated with electroacupuncture in the ST36 acupoint at the right hind paw ipsilaterally. The structure becomes distinct with the treatment time increasing (Figures 4(c)–4(f)), as shown especially in Figure 4(f). This condition means that electroacupuncture can treat adjuvant-induced arthritis. In addition, the OCT can be used to detect the ankle* in vivo*.

**Figure 5:**
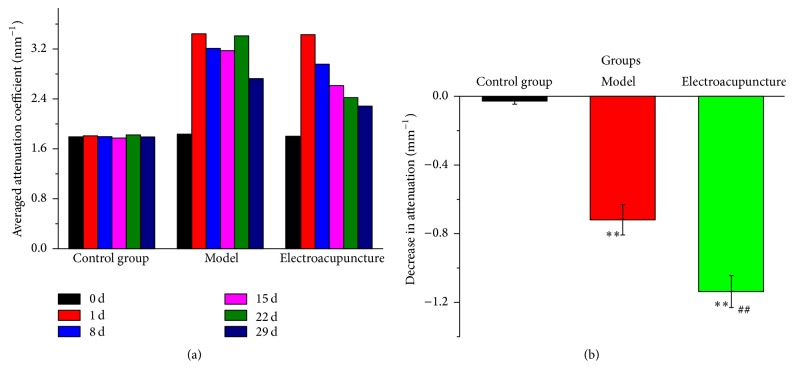
Averaged attenuation coefficients of the ankle of the right hind paws of the rats before modelling (0 day), 1 day after modelling (1st d), 8 days after modelling (8th d), 15 days after modelling (15th d), 22 days after modelling (22nd d), and 29 days after modelling (29th d) from each group (a). The decreased attenuation coefficient of the ankle of the right hind paws of the rats between on 29th d and the 1st d in each group (control, model, and electroacupuncture groups); ^*∗∗*^ *P* < 0.01 in comparison with control group; ^##^ *P* < 0.01 versus EA group at model group (b).

The paper summarises the attenuation coefficients of the ankle of the right hind paws of the rats at 0, 1, 8, 15, 22, and 29 days from the control, model, and electroacupuncture groups, respectively (Figure 5(a)). As seen from Figure 5(a), the light attenuation coefficients of the ankle in the control group did not change with the increase of the time. As also seen from Figure 5(a), comparing day 1 with day 0, the light attenuation coefficients of ankle tissue increased not only in the model group but also in the electroacupuncture group. This increase was observed as a high amount of light was absorbed after the model was established. The light attenuation coefficients of the ankle tissues decreased (e.g., 8 and 15 days) or increased (e.g., 22 days) in the model group because the rats in the model group were not treated after the model was established. In the electroacupuncture group, the light attenuation coefficients of ankle tissues decreased as the time was prolonged after electroacupuncture was administered (Figure 5(a)). These results agree with the obtained images, which showed that adjuvant-induced arthritis can be treated with electroacupuncture stimulating the* “Zusanli”* acupoint. There was decreased attenuation of the ankle of the right hind paws of the rats compared on the 29th day with the 1st day in the control, model, and electroacupuncture groups, respectively (Figure 5(b)). There was a significant difference in attenuation coefficient decrease between the 29th d and the 1st d not only in electroacupuncture group compared with control group, but also in model group compared with control group (*P* < 0.01). It also could be found that there was a significant decrease in the electroacupuncture group compared with the model group (Figure 5(b)). Although the rats with adjuvant-induced arthritis could slowly reduce the swelling as autoimmunity, electroacupuncture group at “Zusanli” could better accelerate the healing of rats with rheumatoid arthritis (consistent with Figure 5 results). Acupuncture or electroacupuncture at “Zusanli” in rats with rheumatoid arthritis could significantly improve local and systemic symptoms, alleviate joint swelling, and prevent inflammatory reactions [35, 36]. According to* “Lingshu”* (Miraculous Pivot), “If the syndrome of Zhuobi (fixed obstruction) does not disappear and its accompanying symptom of long coldness continues, the* “Zusanli”* (ST36) is chosen as the major acupoint to treat the syndrome.”

## 4. Conclusion

Electroacupuncture could be employed to stimulate* “Zusanli”* acupoint and to accelerate treatment of rats with adjuvant-induced arthritis. This study also demonstrated that the OCT could be applied to monitor the treatment effect on the depth of the ankles of rats with adjuvant-induced arthritis* in vivo*. The treatment efficacy of drugs can also be determined by measuring the penetration depth of the ankles of rats through OCT or other strategies.
